# Silicone Sealant as a Urethrovesical Foreign Body: Lessons in Surgical Management

**DOI:** 10.7759/cureus.97087

**Published:** 2025-11-17

**Authors:** Faraz Sharif, Reuben Lai, Angus Hall, Mohamed El-Ghazawy

**Affiliations:** 1 Department of Urology, Mid Yorkshire Teaching NHS Trust, Wakefield, GBR

**Keywords:** bladder foreign body, cystotomy, endourology, silicone sealant, urethral foreign body

## Abstract

We present a rare case of a male patient who presented following self-injection of silicone sealant into a condom placed within the urethra that migrated intravesically. The patient had mild voiding symptoms and haematuria. Imaging demonstrated radio-opaque foreign material extending from the bulbar urethra into the bladder. Endoscopic retrieval was unsuccessful due to the material’s size and consistency. Definitive management was achieved via open Pfannenstiel cystotomy, with intact removal of the foreign body and uneventful recovery. Follow-up cystography and flow rate studies within two months confirmed no complications. Silicone sealant within the lower urinary tract represents an unusual and complex foreign body. This case adds to the limited literature on gathering evidence on optimal management techniques.

## Introduction

Insertion of foreign material into the urethra or bladder is an infrequent but recognised presentation in urology, most commonly associated with autoerotic stimulation, psychiatric illness, or curiosity [1,2]. A wide variety of foreign objects have been documented, including electrical wires, pens, and thermometers [2]. Silicone sealant represents an exceptional example due to its ability to solidify within the urinary tract, creating a rigid mould that complicates removal [3-7]. Such polymerization can cause significant urethral and bladder injury, necessitating precise surgical planning.

Previous reports have shown that endoscopic management of silicone sealant-related cases is rarely successful, with most requiring open surgery for definitive management [3-7]. This report describes a rare instance of silicone sealant migration into the bladder and highlights the operative considerations involved in achieving safe and definitive removal.

## Case presentation

A 25-year-old fit and well male was admitted to the Urology service following self-injection of silicone sealant into a rolled condom placed in the urethra. At the time of insertion, the condom was presumed to have ruptured, resulting in migration of silicone proximally. The patient presented to the hospital 12 hours after initial insertion, reporting symptoms of dysuria, haematuria, and mild straining. However, he was able to void and denied incontinence. Motivation behind the incident was uncertain, with the patient reporting it as ‘a heat of the moment’ decision. He later declined referral to psychological/psychiatric services.

On examination, the abdomen was soft and non-distended, with no peritonitis. Palpation revealed a firm structure within the proximal bulbar urethra. A bladder scan demonstrated 200 mL of retained urine.

**Figure 1:**
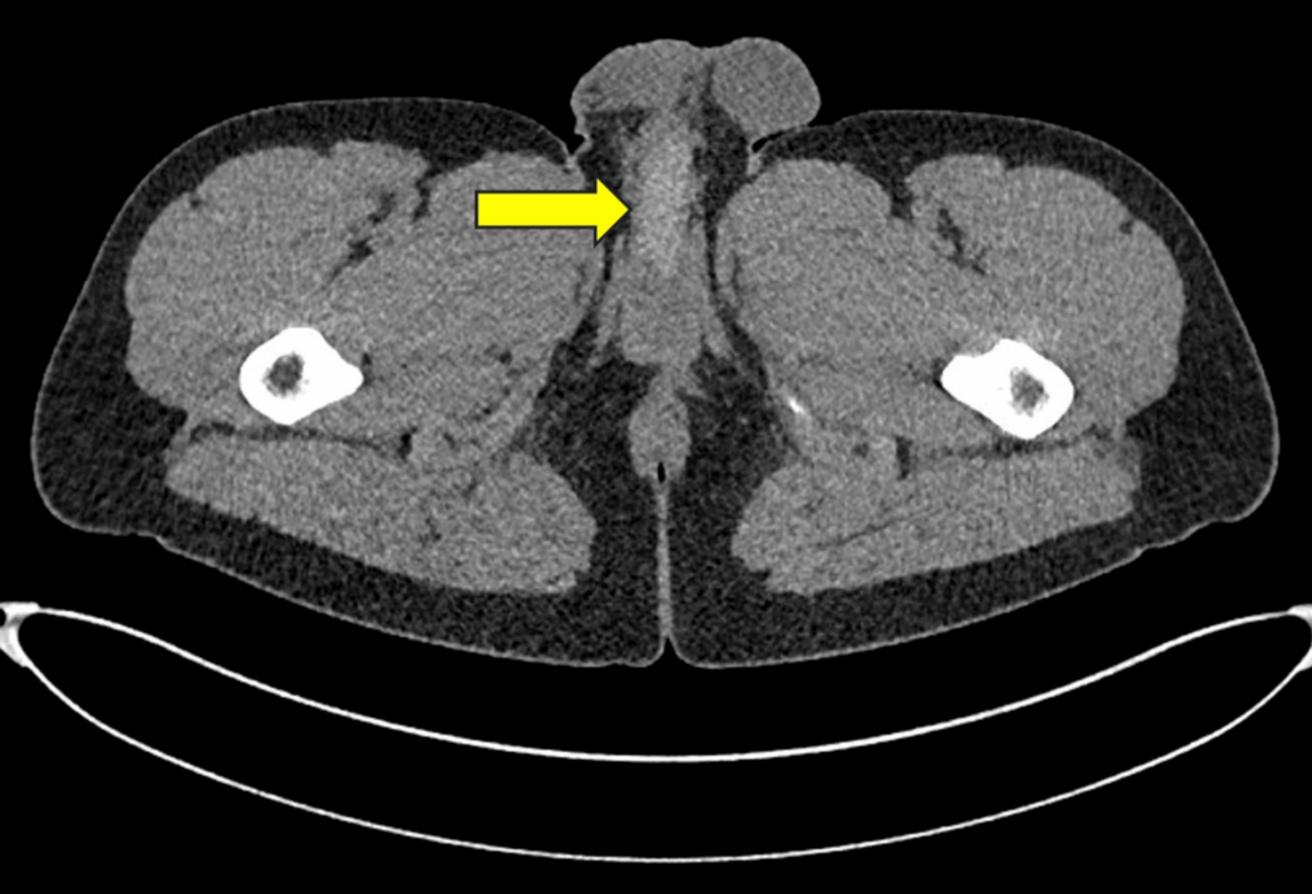
Axial CT image demonstrating a radio-opaque silicone material within the bulbar urethra (yellow arrow).

**Figure 2:**
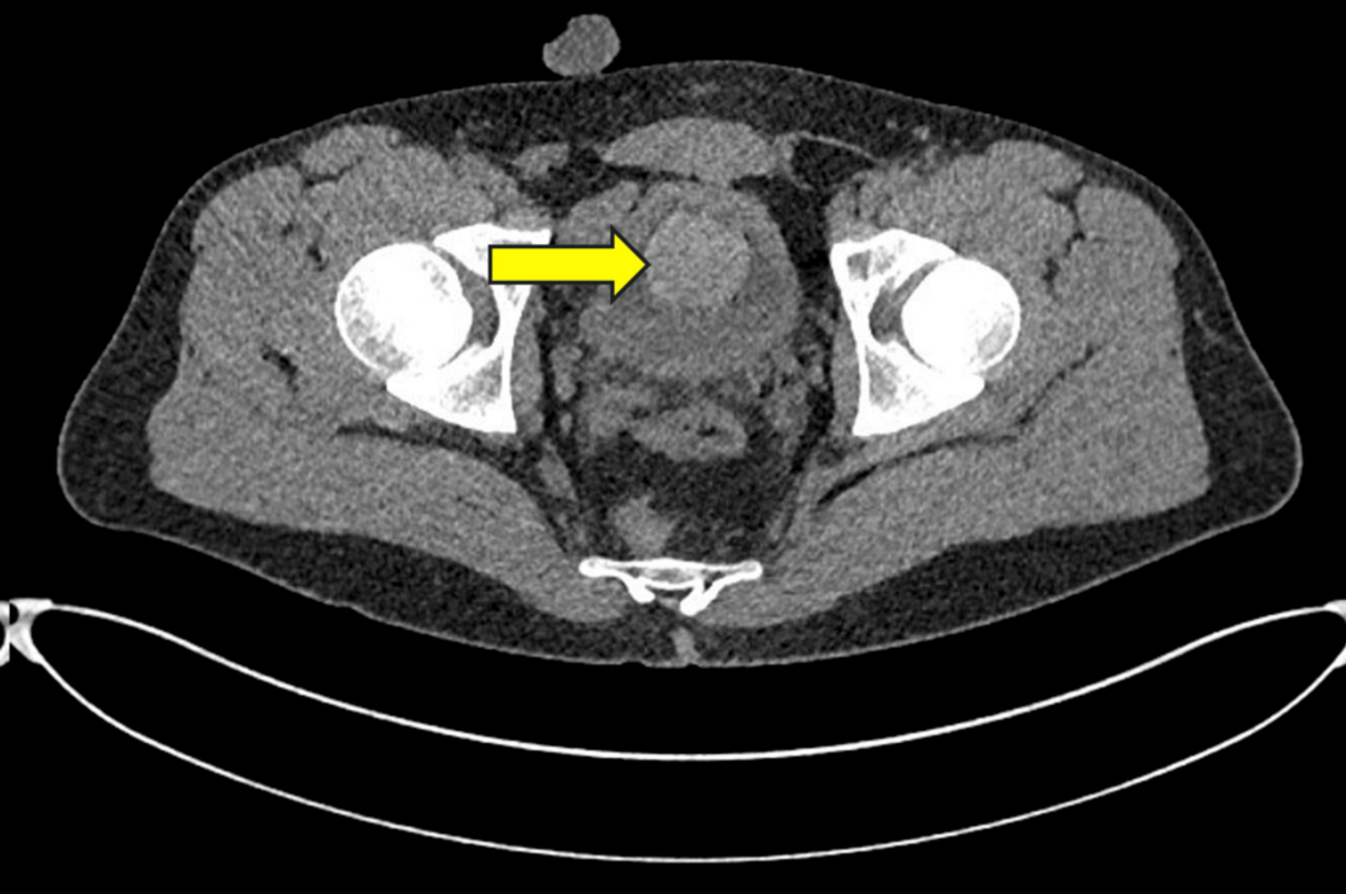
Axial CT image demonstrating a radio-opaque silicone material collected within the bladder (yellow arrow).

Emergency ambulatory flexible cystoscopy revealed a foreign body extending from the bulbar urethra, with inability to advance a guidewire beyond the obstruction. The material was thick and plastered to the urethral wall. It was graspable but not retrievable. The procedure was abandoned, and a CT scan of the pelvis was conducted to assess the extent of the material. This confirmed an intact, continuous radio-opaque foreign material extending from the bulbar urethra to the bladder, with a large ball of the material present in the bladder (Figures 1-2). 

**Figure 3:**
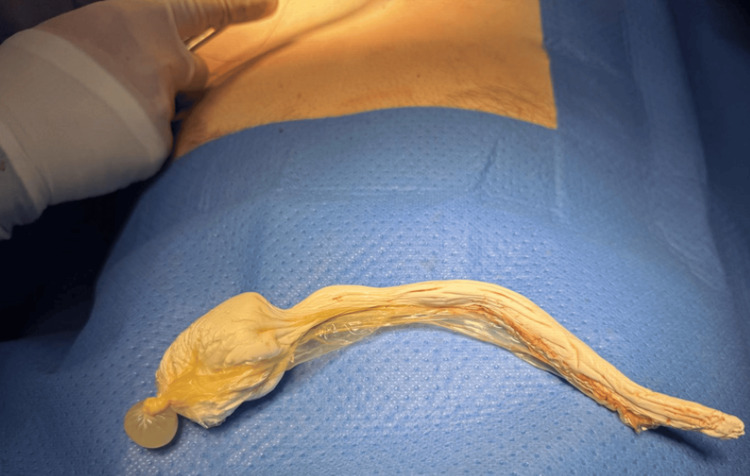
Silicone foreign body removed intact after cystotomy, encased within the condom, measuring at 18 cm across.

Given failed endoscopic management and the size, consistency, and location of the material, the patient underwent laparotomy with Pfannenstiel cystotomy. The bladder was opened transversely. The foreign body was removed with ease, coming out in a perfect mold of the bladder and proximal urethra and encased by the condom (Figure 3). A 14Fr urethral catheter was placed. The bladder was closed in two layers with 2-0 Vicryl, and a watertight closure was confirmed with an intraoperative leak test.

The postoperative course was uneventful. The patient was discharged home the next day with a catheter in situ, declining psychiatric referral prior to discharge. A cystogram performed at three weeks demonstrated no extravasation. Catheter removal was followed by normal voiding. At two-month follow-up, the patient was asymptomatic, and flow studies showed a peak flow rate of 39.2 mL/s and a post-void residual of 35 mL.

## Discussion

Self-insertion of urethral foreign bodies remains a rare but recognized phenomenon, often motivated by autoerotic stimulation, psychiatric illness, or experimentation [1]. While a variety of objects have been reported, silicone sealant is rare and is particularly problematic due to its ability to polymerize in situ, forming a rigid, space-occupying cast that conforms tightly to the urethral and bladder lumen [2].

Previous cases have described patients either presenting after failed removal or in acute urinary retention with common symptoms including dysuria, haematuria, and voiding symptoms [3-7]. All cases noted in the literature have necessitated open procedures to remove the sealant via either cystostomy or urethrotomy [3-7]. This has been irrespective of size, with the lengths of foreign bodies reported in literature ranging from 10-15 cm, compared to the 18 cm in this study [3-7]. All endoscopic attempts to remove the sealant failed [4-7]. Our case adds to this evidence, with endoscopic management being noted to be difficult, likely due to the composition of the sealant and its intravascular component preventing urethral removal.

Management should be dictated by the material’s location and size. Palmer et al. proposed an algorithm for the management of urethral foreign bodies, which suggests that, if the object is larger than 1 cm, non-palpable, immobile, or proximal to the distal penile urethra, then imaging should be conducted [8]. This would allow assessment of any bladder component of the object, which could help guide whether endoscopic management or open management would be more suitable. In our case, a decision was made to trial endoscopic management due to only partial obstruction, patient preference, and to avoid unnecessary invasive surgery. However, after failure of endoscopic management and due to the extent of the foreign material, a decision was made to proceed with the open approach.

Alternative minimally invasive strategies, such as endoscopic fragmentation or laser ablation, were not considered due to the degree of spread into the bladder and the difficulty of access urethrally. Bedke et al. suggest that most medical and non-medical objects can be broken down by laser [9], although Mustafa et al. reported that laser fragmentation failed on silicon [4]. Open cystotomy ensured complete extraction with preservation of urethral integrity. Postoperative outcomes, with a resolution of symptoms and normal flow studies, highlight the safety of this approach.

In terms of complications, there were several reports of patients developing urethral strictures post-operatively [5-7]. Time to intervention for these patients ranged from approximately seven hours to one month. Meanwhile, Trishch et al. and Mostafa et al. reported patients who had definitive intervention at one and two weeks after initial insertion who did not have any complications [3,4]. This shows that it is inconclusive whether time to intervention is significant to prevent complications. More likely, complications will be due to the effects of the chemical properties of sealants on the urethral mucosa, with a high risk of urethral strictures and urinary tract infections. Our patient had the good fortune to have his urethral mucosa partially protected by the condom, which may have contributed to his satisfactory outcome.

## Conclusions

Self-insertion of urethral foreign bodies is rare, but injection of sealant intra-urethrally is even less common and poses more challenges. Our case adds to the limited literature showing that endoscopic management does not tend to work for this patient group. When endoscopic removal is not feasible, open cystotomy has provided a definitive and safe solution, with satisfactory functional recovery. More data are required on similar cases and outcomes to help guide a standardized framework for management.
